# Red edge excitation shift spectroscopy is highly sensitive to tryptophan composition

**DOI:** 10.1098/rsif.2023.0337

**Published:** 2023-11-08

**Authors:** Annmaree K. Warrender, Jolyn Pan, Chris Pudney, Vickery L. Arcus, William Kelton

**Affiliations:** ^1^ Te Huataki Waiora School of Health, University of Waikato, Hamilton, New Zealand; ^2^ Te Aka Mātuatua School of Science, University of Waikato, Hamilton, New Zealand; ^3^ Department of Biology and Biochemistry, University of Bath, Bath, UK

**Keywords:** red edge excitation shift, protein dynamics, tryptophan fluorescence, protein stability, antibodies

## Abstract

Red edge excitation shift (REES) spectroscopy relies on the unique emission profiles of fluorophore–solvent interactions to profile protein molecular dynamics. Recently, we reported the use of REES to compare the stability of 32 polymorphic IgG antibodies natively containing tryptophan reporter fluorophores. Here, we expand on this work to investigate the sensitivity of REES to variations in tryptophan content using a subset of IgG3 antibodies containing arginine to tryptophan polymorphisms. Structural analysis revealed that the additional tryptophan residues were situated in highly solvated environments. Subsequently, REES showed clear differences in fluorescence emission profiles when compared with the unmutated variants, thereby limiting direct comparison of their structural dynamics. These findings highlight the exquisite sensitivity of REES to minor variations in protein structure and tryptophan composition.

## 1. Introduction

The intrinsic red shift properties of tryptophan (Trp) fluorescence have proven useful for evaluating protein structural dynamics and stability. Termed red edge excitation shift (REES), it has been nearly 40 years since the correlation was first made between Trp fluorescence emission spectra and the local solvation environment within proteins [1–5]. Since then, a number of models have been proposed to relate the REES effect to protein molecular dynamics. These models have been useful for monitoring protein stability and plasticity [6–11], understanding mechanism of ligand binding [12,13] and linking enzyme kinetics to conformational flexibility [14].

Measurement of the REES effect via spectroscopy involves the quantification of Trp emission at progressively longer excitation wavelengths, sampling unique Trp microstates with each step [15]. The overall change in maximum emission energy across excitation energies is dependent on the ensemble of Trp microenvironments, which are affected by the physical and structural parameters of the sample such as polarity, packing density, solvent accessibility and exciplex formation [1,2,16,17]. In proteins, changes to the microenvironments occur due to subtle fluctuations in the folded structure or from larger structural rearrangements during unfolding or aggregation events [18,19]. Interactions between Trp and surrounding molecules during fluorescent excitation influence the time scale of fluorescent relaxation and emission [4,17,20]. While the technique is experimentally simple, requiring only a laboratory-grade spectrophotometer, the mechanistic underpinnings are less so, and it is worth taking time to understand the REES phenomena to support interpretation of the resulting spectra.

**Figure 1.**
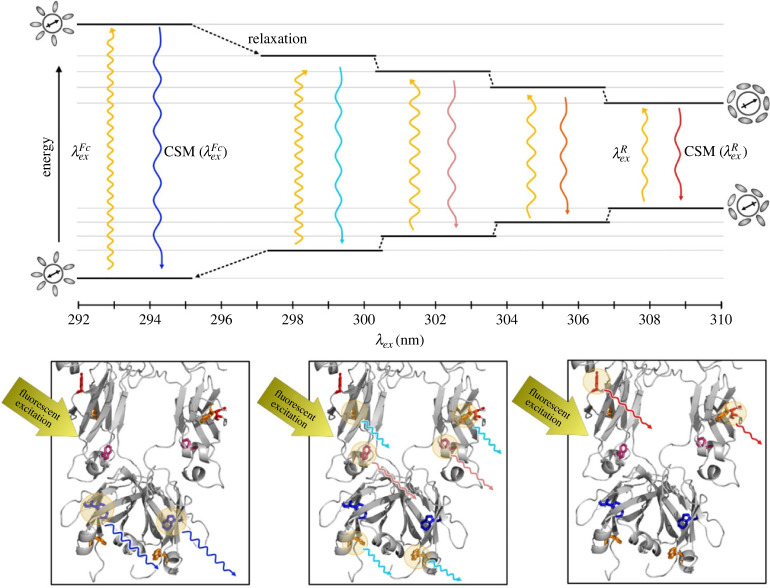
Depiction of the REES phenomenon in relation to excitation and emission energies. (Top) A Jablonski diagram showing energy transitions between the excited and relaxed states of Trp and the corresponding energy of emission. The circles containing arrows represent the dipole moment of Trp with orientation of the surrounding solvent molecules depicted by ovals. As Trp is excited, the dipole moment changes and the energy increases. As the solvent molecules reorientate around the new dipole moment, the energy decreases. The rate of relaxation relative to the fluorescence lifetime determines the transition state energy at which fluorescence emission occurs. As the energy of emission decreases, the ground state is of higher energy, which is readily excited by the subsequent lower excitation energies. (Bottom) Trp residues with unique solvation states are represented in part of the antibody structure to demonstrate the contribution of different Trp to emission energy. From left-to-right, blue Trp (low solvent exposure, rigid environment) emit from high energy, orange Trp (mid-range solvent exposure) emit from slightly lower energy, pink Trp (some solvent exposure) emit from low energy, and red Trp (highly solvent exposed, flexible environment) have the most red-shifted emission.

REES is underpinned by the interaction of polar solvent molecules (water) with Trp as they are excited by light within the UV range of the spectrum [1,2,21–23]. The absorption of a photon by Trp induces a high energy state as a result of energy transfer to both the electronic and vibrational degrees of freedom of the fluorophore (figure 1). This process is accompanied by a characteristic change in the dipole moment of Trp. The surrounding polar solvent molecules reorientate to accommodate the altered dipole moment and reach an energetically favourable state. These time scales are heavily dependent on the polarity of the surrounding solvent and the number of possible discrete solvent orientation states to be sampled [1,2,15,23]. In any given protein with multiple Trp residues, each position will contribute differently to the fluorescent emission profile.

For highly flexible or unstructured protein regions, where Trp solvent accessibility is high, the reorientation of the water molecules occurs relatively quickly compared with Trp fluorescence lifetime [15]. There are few interactions with the surrounding protein environment to impede the movement of water molecules surrounding the fluorophore. Therefore, the observed fluorescence emission occurs entirely from a low-energy solvent relaxed state and does so independently of the excitation wavelength [7,15,24,25]. Only a minimal red-edge effect is observed in this case (figure 1). In rigid and structured regions of proteins with buried Trp residues, there can be significant interaction with the surrounding protein microenvironment. As a result, reorganization of water molecules around a recently excited Trp are significantly slower than in unstructured protein regions [26]. In this instance, the time taken for surrounding solvent molecules to reorientate to the more favourable, lower energy state exceeds the fluorescent lifetime, resulting in emission from the excited, high-energy state [17,25]. In systems with multiple Trp residues, excitation with progressively lower energy quanta (longer excitation wavelengths) specifically selects for the subpopulation of Trp residues requiring less energy to excite [15,26]. As shown in figure 1, this population of Trp residues have a higher energy ground state (as the solvent molecules have not yet reorientated to the Franck–Condon (FC) ground state) and a corresponding lower energy excited state. It is the specific excitation of these Trp residues that gives rise to the REES effect and provides an overall estimate of the solvent accessibility within a protein [2,23].

One complication arising from the use of the Trp REES effect to predict protein flexibility is the occurrence of three excited rotamer states available to Trp sidechains that exert unique red-edge effects of emission [12,20,27,28]. The excited dipole moment can exist in unique orientations that hold different interaction energies with the surrounding molecules which affect the relaxation time and fluorescent emission energies [22,29]. Contributions from each Trp residue in each rotamer formation will influence the overall REES effect. Therefore, to implement REES for the biophysical comparison of multiple proteins, the number and position of Trp residues must be considered. To ensure differences can be attributed to the structural behaviour of the protein backbone, it has been suggested that proteins should have the same number of Trp residues positioned in similar microenvironments [8,12]. However, there has been little experimental data gathered to determine how influential such differences might be on REES spectra.

**Figure 2.**
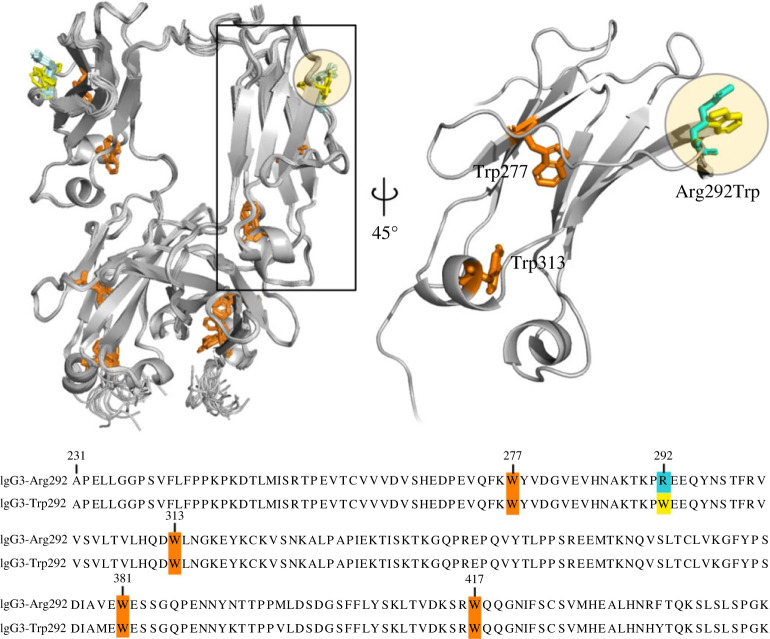
Structure and sequence alignment of IgG3 variants. (Top left) Overlaid structures of the Fc region (CH2-CH3) of all IgG3 alleles showing the location of conserved Trp (orange sticks), Arg292 (cyan sticks) and Trp292 (yellow sticks). (Top right) A single CH2 domain highlighting the position of the Arg292Trp polymorphism. (Bottom) Sequence alignment of IgG3-Arg292 and IgG3-Trp292 Fc region only, highlighting the conserved Trp (W, orange), Arg292 (R, cyan), and Trp292 (yellow). Sequences given are for IgG3*01 and IgG3*18 alleles, respectively, as representative sequences. Residues are numbered based on the EU numbering system.

Here, we have extended a prior REES analysis of 32 highly similar monoclonal antibodies to include Trp variation [30] and demonstrated the technique is highly sensitive to Trp residue content and sequence position. We measured IgG3 antibodies with identical Fab domains and unique Fc sequences containing human allele-encoded amino acid polymorphisms [31]. While most of the Trp residues are highly conserved throughout the Fc sequences, three of the variants carry an arginine (Arg) to Trp mutation at position 292 resulting in two additional Trp residues (given that antibody heavy chains are homodimers) located on the outer edge of the antibody structure (figure 2). We found a significant difference in REES effect between the IgG3-Arg292 variants compared with IgG3-Trp292 variants. Our study demonstrates the importance of considering Trp content and positioning within the protein structure when using REES for comparative analysis.

## 2. Methods

### 2.1. Antibody selection and expression

Antibodies were cloned, expressed and purified as described previously [30]. In brief, trastuzumab (Herceptin) variable domain sequences were used for the heavy and light chain variable domains (electronic supplementary material, table S1). The constant domain sequences were obtained from The International Immunogenetics Information System (IMGT^Ⓡ^) database [32]. For the light chain of all antibodies, the IGKC*01 kappa constant allele was selected and appended to the trastuzumab light chain variable domain sequence with a rabbit IGKC signal peptide in the expression vector, pTwist CMV BetaGlobulin WPRE Neo (Twist Biosciences, USA). The heavy chain constant domain sequences for each human IgG3 allele were appended to the trastuzumab heavy chain variable sequence with a rabbit IGHG signal sequence. Antibodies were expressed using the Expi293 expression system (Thermo Fisher, USA) and purified using Protein G Sepharose (Thermo Fisher, USA).

### 2.2. Homology modelling and sequence alignment

Homology models of the antibodies were generated using Robetta (https://robetta.bakerlab.org/), a Rosetta-based protein structure prediction software. Sequences of each of the IgG3 anti-HER2 antibodies were threaded onto the template structure of full length IgG1 anti-gp120 mAb (PDB: 1HZH). The predicted models were validated using Molprobity to generate Ramachandran plots [33] and the Fc regions were aligned to the human IgG3 Fc structure (5W38) using PyMOL to determine root-mean-square deviation (RMSD) values [34]. Sequence alignments were performed using Geneious Pairwise Alignment in Geneious Prime 2020.0.5.

### 2.3. Solvent accessibility calculations

The homology models were used to calculate the relative percentage of solvent accessibility of each Trp residue for each structure in PyMOL. The total solvent accessible surface area (SASA) was calculated for all Trp residues within a structure and divided by the number of Trp residues (22 for IgG3-Arg292 variants, 24 for IgG3-Trp292 variants) to give the average SASA per Trp across the molecule, in angstrom squared (Å^2^).

### 2.4. Red edge excitation shift analysis

REES data collection and analysis were performed using the methods reported by Warrender *et al.* [30]. Antibody samples were diluted to 0.15 mg ml^−1^ with 50 mM Tris-HCl buffered saline pH 8.0. Samples were measured in triplicate in magnetically stirred cuvettes at 10°C in a temperature-controlled chamber in a Hitachi F-7000 fluorescent spectrometer. Fluorescent scans were performed by exciting at wavelengths from 292 to 310 nm and monitoring emission wavelengths between 325 and 500 nm, increasing in 1 nm steps. Slit widths were set to 5 nm.

Fluorescent data was processed by first applying equation (2.1) to determine the centre of spectral mass (CSM) for each sample. Where *f_i_* is the measured fluorescence intensity and *λ*_Em_ is the emission wavelength.

2.1 $$\mathrm{CSM}=\frac{\Sigma (\;f_{i}\lambda_{\mathrm{Em}})}{\Sigma (\;f_{i})}.$$

Subsequently, the CSM data was modelled using equation (2.2) [35].

2.2 $$\mathrm{CSM}(\lambda_{\mathrm{Ex}})=\;\;\frac{\mathrm{CSM}(\lambda_{\mathrm{Ex}}^{Fc})+\mathrm{CSM}(\lambda_{\mathrm{Ex}}^{R})e^{\left(m(\lambda_{\mathrm{Ex}}-\lambda_{\mathrm{Ex}}^{50\text{\%}})/RT\right)}}{1+e^{\left(m(\lambda_{\mathrm{Ex}}-\lambda_{\mathrm{Ex}}^{50\text{\%}})/RT\right)}}.$$

The CSM of the relaxed state, $\mathrm{CSM}(\lambda_{Ex}^{R})$, is a theoretical measure of the emission wavelength if the antibodies were fully solvated. This is dependent on the number of tryptophan residues and therefore would be the same for each antibody provided the tryptophan content is the same [35]. The antibodies tested were classified into two groups, denoted IgG3-Arg292 and IgG3-Trp292, based on the number of tryptophan residues contained within the sequence (22 or 24, respectively) and treated separately for the model fitting process. $\mathrm{CSM}(\lambda_{Ex}^{R})$ was globally fit within each group, with constraints set between 387 and 440 nm to align with a practical range determined previously [35]. The constant variables, R (gas constant, 8.3145 J mol^−1^ K^−1^), T (temperature, 283.15 K) and $\lambda_{Ex}$ (excitation wavelength, 292–310 nm), were kept the same for each sample, regardless of Trp content. The remaining variables were unique to each sample and determined using a nonlinear regression. For context, $\mathrm{CSM}(\lambda_{\mathrm{Ex}}^{Fc})$ is the maximum wavelength of emission from a fully excited (Franck–Condon) state, $\lambda_{\mathrm{Ex}}^{50\text{\%}}$ is the excitation wavelength at the CSM halfway between $\mathrm{CSM}(\lambda_{Ex}^{Fc})$ and $\mathrm{CSM}(\lambda_{\mathrm{Ex}}^{R})$ (the inflection point of the sigmoid), and *m* is the gradient of the slope, reflecting the change in Gibbs free energy (ΔG) as a function of $\lambda_{Ex}$. In the text, the value of *m* is referred to as ΔG*_m_* with units of J mol^−1^ nm^−1^.

Statistical analyses were performed using one-way ANOVA testing and Tukey *post hoc* multiple comparisons tests. The *p*-adjusted values are reported with a significance cut-off of *p* = 0.05.

## 3. Results

### 3.1. IgG Fc variants with differences in the number of tryptophan residues exhibit differing red edge excitation shift effects

REES data were collected for 21 trastuzumab-formatted antibodies with unique IgG3 constant domains. Of these variants, 18 contained 22 Trp residues (IgG3-Arg292 variants) and three contained 24 Trp residues (IgG3-Trp292 variants).

**Figure 3.**
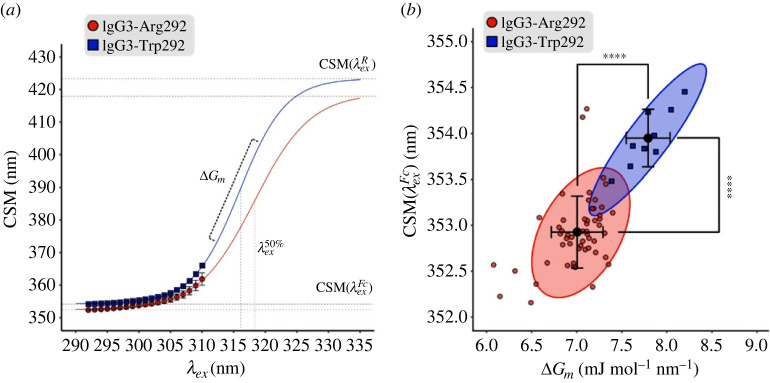
Processed REES data comparing antibody groups. (*a*) CSM profiles for IgG3-Arg292 (variant IgG3*17, red circles) and IgG3-Trp292 (variant IgG3*19, blue squares) fitted with the sigmoidal REES model from equation (2.2). The data points represent the average of three replicate measurements with error bars showing standard deviation. Dotted lines show the values of $\mathrm{CSM}(\lambda_{\mathrm{Ex}}^{R})$, $\mathrm{CSM}(\lambda_{Ex}^{Fc})$, ΔG_m_ and $\lambda_{\mathrm{Ex}}^{50\text{\%}}$ derived from the model. (*b*) Derived REES values of $\mathrm{CSM}(\lambda_{Ex}^{Fc})$ plotted against ΔG_m_ for all IgG3 variants. Data points represent each measured replicate of IgG3-Arg292 variants (red circles) and IgG3-Trp292 variants (blue squares). A 95% data ellipse shows the spread of data for each group. The black circle in the centre of each ellipse represents the mean for each group with error bars showing the standard deviation. Significance levels were tested using one-way ANOVA with Tukey *post hoc* multiple comparison testing. The difference between $\mathrm{CSM}(\lambda_{Ex}^{Fc})$ (top) and ΔG_m_ (right side) between the antibody groups is shown to be of high significance. * *p* < 0.05, ** *p* < 0.01, *** *p* < 0.001, **** *p* < 0.0001.

REES parameters were derived from the fluorescence emission profiles by fitting the data with a recently established thermodynamic model [31] (equation (2.2), electronic supplementary material, figure S2). Using this model, the solvation state of the ensemble of tryptophans (described by $\mathrm{CSM}(\lambda_{\mathrm{Ex}}^{\mathrm{Fc}})$) and the overall flexibility of the globular protein structure (described by ΔG*_m_*) can be inferred (figure 3*a*). Higher values of $\mathrm{CSM}(\lambda_{\mathrm{Ex}}^{\mathrm{Fc}})$ (the centre of spectral mass of the fully excited Franck–Condon state) indicate more Trp residues are in a solvent-exposed state and therefore emitting at a lower energy. Likewise, higher values of ΔG*_m_* (related to the number of solvent–fluorophore interactions) indicate greater protein rigidity. Finally, the model derives $\mathrm{CSM}(\lambda_{\mathrm{Ex}}^{R})$ (the centre of spectral mass of the completely relaxed state), which is assumed to be similar for highly similar protein structures with the same number of Trp residues and represents the predicted fluorescence of Trp in a completely unfolded state with full solvent access. It is not possible to measure $\mathrm{CSM}(\lambda_{\mathrm{Ex}}^{R})$ experimentally using conventional spectrophotometers as there is generally insufficient UV light intensity at longer, low-energy wavelengths (*λ_Ex_* > 310 nm) to generate signal above the noise. To accurately derive this value from the model, $\mathrm{CSM}(\lambda_{\mathrm{Ex}}^{R})$ was fit globally for antibody samples of the same group (IgG3-Arg292 or IgG3-Trp292), resulting in values of 418.9 ± 2.9 nm for IgG3-Arg292 variants and 423.3 ± 16.2 nm for IgG3-Trp292 variants (figure 3*a*, table 1).

**Table 1.** Values of $\mathrm{CSM}(\lambda_{Ex}^{Fc})$, ΔG_m_ and $\mathrm{CSM}(\lambda_{Ex}^{R})$ derived from fitting the fluorescence emission centre of spectral mass profiles with the sigmoidal REES model. Values given are the average (avg) of triplicate measurements with standard deviations (s.d.). SASA per Trp calculated using PyMOL is given for each variant. The group averages for IgG3-Arg292 and IgG3-Trp292 are shown in the bottom section.

		$\mathrm{CSM}(\lambda_{Ex}^{Fc})$		ΔG*_m_*		$\mathrm{CSM}(\lambda_{Ex}^{R})$		average SASA per Trp (Å^2^)	
	allele	avg	s.d.	avg	s.d.	avg	**s.d.**	avg	sd
IgG3-Arg292	3*01	353.1	±0.2	7.0	±0.2	418.9	±2.9	4.89	
	3*03	352.5	±0.2	7.3	±0.1	418.9	±2.9	4.75	
	3*04	353.1	±0.2	7.1	±0.2	418.9	±2.9	7.12	
	3*06	353.1	±0.9	6.9	±0.3	418.9	±2.9	6.87	
	3*08	352.7	±0.2	6.6	±0.5	418.9	±2.9	6.85	
	3*09	352.9	±0.0	7.0	±0.1	418.9	±2.9	10.73	
	3*11	352.9	±0.1	7.0	±0.1	418.9	±2.9	6.63	
	3*12	352.7	±0.4	6.7	±0.5	418.9	±2.9	8.69	
	3*13	352.7	±0.2	7.1	±0.2	418.9	±2.9	10.52	
	3*14	353.0	±0.4	7.1	±0.1	418.9	±2.9	8.67	
	3*15	353.1	±0.4	7.2	±0.1	418.9	±2.9	8.86	
	3*16	353.6	±0.6	7.2	±0.1	418.9	±2.9	11.49	
	3*17	352.4	±0.2	6.6	±0.3	418.9	±2.9	8.86	
	3*20	353.2	±0.2	6.8	±0.3	418.9	±2.9	8.45	
	3*22	352.9	±0.3	6.9	±0.3	418.9	±2.9	7.07	
	3*24	352.9	±0.2	7.1	±0.1	418.9	±2.9	6.93	
	3*25	353.1	±0.1	7.3	±0.1	418.9	±2.9	8.06	
	3*26	352.7	±0.1	7.1	±0.1	418.9	±2.9	8.75	
IgG3-Trp292	3*18	353.7	±0.2	7.7	±0.3	423.3	±16.2	21.27	
	3*19	354.2	±0.2	8.0	±0.2	423.3	±16.2	15.91	
	3*23	353.9	±0.3	7.7	±0.1	423.3	±16.2	19.24	
	IgG3-Arg292	352.9	±0.3	7.0	±0.2	418.9	±2.9	8.01	±1.77
	IgG3-Trp292	353.9	±0.3	7.8	±0.2	423.3	±16.2	18.81	±2.21

The values of $\mathrm{CSM}(\lambda_{\mathrm{Ex}}^{\mathrm{Fc}})$ and ΔG*_m_* were determined for each individual sample and subsequently grouped for data analysis (table 1). The $\mathrm{CSM}(\lambda_{\mathrm{Ex}}^{\mathrm{Fc}})$ values of IgG3-Arg292 variants varied between 352.4 and 353.6 nm with an average of 352.9 ± 0.3 nm (figure 3*b*). The values for IgG3-Trp292 variants were significantly higher (*p* < 0.001) with a minimum $\mathrm{CSM}(\lambda_{\mathrm{Ex}}^{\mathrm{Fc}})$ of 353.7 nm, maximum of 354.2 nm and average of 353.9 ± 0.3 nm. The same trend was evident for ΔG*_m_*, for which IgG3-Trp292 variants exhibited significantly higher (*p* < 0.001) values between 7.7 and 8.0 mJ mol^−1^ nm^−1^ with an average of 7.8 ± 0.2 mJ mol^−1^ nm^−1^ while IgG3-Arg292 variants had ΔG*_m_* values between 6.6 and 7.3 mJ mol^−1^ nm^−1^ with an average of 7.0 ± 0.2 mJ mol^−1^ nm^−1^ (figure 3*b*, table 1).

### 3.2. Arg292Trp mutations in antibody constant regions significantly increase the average solvent accessible surface area of tryptophan residues

To characterize the solvent accessibility of the Trp microenvironments within the antibody structures, PyMOL was used to predict the percentage of relative solvent exposure of each Trp residue in modelled Fc domains. Model statistics for each allele are provided in electronic supplementary material, table S2 and figure S1 with most RMSD values less than 3 Å relative to the human IgG3 Fc structure 5W38. We note this structure is missing the hinge region and is in a more open configuration than the full-length structure used for threading. In this analysis, 0% indicates a residue with complete solvent inaccessibility (such as for Trp located tightly within the protein folds) and 100%, a fully solvent exposed residue. It should be noted that antibody heavy chains are homodimeric, and therefore the Arg292Trp mutation results in a net increase of two Trp per molecule. Despite this heavy chain symmetry, the estimated percentage of solvent exposure was not necessarily equal for each copy of Trp residue (electronic supplementary material, table S3). This can be attributed to the homology-modelled structures capturing Trp sidechains in different rotamer states on each chain. The additional Trp residues in IgG3-Trp292 variants had predicted levels of solvent exposure between 33% and 62%. This was substantially greater than the highest exposed Trp found in IgG3-Arg292 variants, with an estimated solvent exposure of 29%.

Subsequently, we estimated the SASA for each Trp residue and calculated the average SASA per Trp for each antibody variant (table 1). We observed a higher average SASA per Trp of 18.8 ± 2.2 Å^2^ for IgG3-Trp292 variants compared with just 8.0 ± 1.8 Å^2^ for IgG3-Arg292 variants.

### 3.3. Relationship between solvent accessible surface area and the red edge excitation shift effect

**Figure 4.**
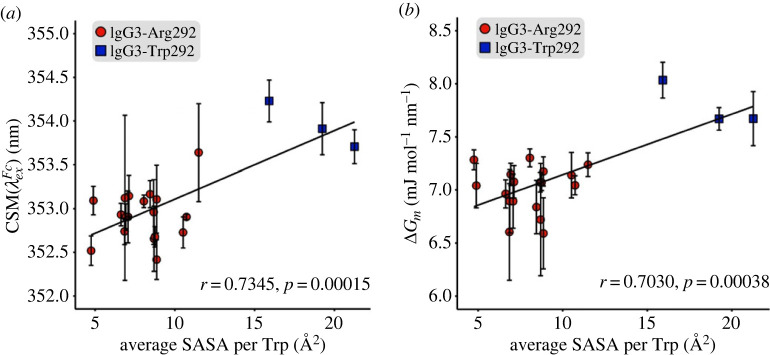
Correlation between average SASA per Trp and (*a*) $\mathrm{CSM}(\lambda_{Ex}^{Fc})$ (*b*) ΔG_m_. Data points represent the average of triplicate measurements for IgG3-Arg292 (red circles) and IgG3-Trp292 (blue squares) variants with error bars to show standard deviation. Solid line shows the line of correlation corresponding with the Pearson correlation coefficient (*r*) and *p*-value.

Pearson correlation coefficients were determined to analyse the relationship between the REES parameter for solvent exposure, $\mathrm{CSM}(\lambda_{\mathrm{Ex}}^{\mathrm{Fc}})$, and the average SASA per Trp residue. A positive correlation was observed with a coefficient of *r* = 0.7345 (*p* < 0.001) (figure 4*a*)*.* We repeated this analysis with the REES parameter for protein rigidity, ΔG*_m_*, and also found a strong correlation of *r* = 0.7030 (*p* < 0.001) (figure 4*b*).

## 4. Discussion

Red edge excitation shift spectroscopy exploits the intrinsic properties of fluorescent reporters such as Tryptophan to assess the structural rigidity and stability of highly similar proteins. We have shown previously that REES is sensitive enough to detect subtle differences in stability between human IgG antibodies with single amino acid differences in the heavy chain [30]. Here, we have expanded upon this analysis to demonstrate the sensitivity of the REES technique to Trp content. Moreover, we provide experimental evidence to support previous suggestions that equivalent Trp content is a prerequisite for comparing protein stability using REES.

A key advantage of the REES technique is the ability to sample a wide range of dynamic protein conformational states in solution to characterize protein flexibility and unfolding, while only requiring conventional laboratory spectrophotometry equipment. Yet, beneath the simplicity of data collection lies the more intricate task of interpreting the acquired data. This analysis is primarily complicated by the fact that REES signal stems from the average fluorescence emission arising from the ensemble of Trp residues throughout the structure. Specifically, Trp residues in different positions can result in unique emission profiles regardless of structural flexibility differences. We used a panel of 21 human IgG3 antibodies which possessed minor differences in Trp composition (22 or 24 Trp residues) alongside additional amino acid polymorphisms (as many as eight mutations between variants). To detangle the influence of Trp composition changes we compared the relative solvent exposure of each Trp residue within the antibody structures. It was evident that the additional two Trp residues present in the IgG3 variants carrying the Arg292Trp mutation had a much larger degree of solvation compared with all other Trp (electronic supplementary material, figure S3). This subsequently increased the SASA per Trp when averaged across all the Trp residues within the structures. We acknowledge that the estimated SASA values are calculated from homology models predicted by threading onto a static IgG1 crystal structure and therefore may differ from dynamic SASA values of these proteins in solution. Nonetheless, our finding of a positive correlation between the increased SASA per Trp and an increase in $\mathrm{CSM}(\lambda_{\mathrm{Ex}}^{\mathrm{Fc}})$ value aligns with previously published data [35]. The disparity observed in $\mathrm{CSM}(\lambda_{\mathrm{Ex}}^{\mathrm{Fc}})$ between the antibody groups with varying Trp content was significantly larger than the variability between variants with an equal number of Trp residues. This finding strongly suggests that the additional Trp residues are probably responsible for the distinct REES profiles observed.

Perhaps counterintuitively, we also report a positive correlation between SASA per Trp and ΔG_m_ (a measure of structural rigidity). When interpreting REES profiles, it is expected that a greater value of ΔG_m_ is indicative of higher structural rigidity. Kwok *et al.* [35] undertook a REES analysis of several engineered ɑ-synuclein protein variants containing single Trp residues at specific locations. They proposed that differences in ΔG_m_ between variants arose from local structural arrangements or from flanking amino acids contributing to solvent–fluorophore interaction energies [35]. In these scenarios, a change in ΔG_m_ will be the result of local microenvironment changes rather than global structural rigidity differences. We suggest this phenomenon explains why we see such an unexpected increase in ΔG_m_ for IgG3-Trp292 variants compared with the IgG3-Arg292 variants.

These results highlight the remarkable sensitivity of REES spectroscopy to fine alterations in Trp solvation states. Although currently underutilized, REES has applications in protein biochemistry and biotechnology, not least of which is the rapid interrogation of large numbers of antibody variants as demonstrated here. However, caution must be taken to ensure the number of Trp residues remains consistent among the proteins being compared. Additional emission signals from extra Trp residues can confound the results and mask any effects stemming from structural disparities in the proteins.

## Data Availability

We have provided raw REES data plots and model fits in the supplementary information. The data are provided in electronic supplementary material [36].
